# Prognostic Impact of Left Ventricular Ejection Fraction Improvement after Transcatheter Aortic Valve Replacement

**DOI:** 10.3390/jcm13133639

**Published:** 2024-06-21

**Authors:** Jakob Johannes Reichl, Thorald Stolte, Shihui Tang, Jasper Boeddinghaus, Max Wagener, Gregor Leibundgut, Christoph Ado Kaiser, Thomas Nestelberger

**Affiliations:** 1 Department of Cardiology and Cardiovascular Research Institute Basel (CRIB), University Hospital Basel, University of Basel, 4031 Basel, Switzerland; jakobjohannes.reichl@usb.ch (J.J.R.); thorald.stolte@usb.ch (T.S.); shihui.tang@usb.ch (S.T.); jasper.boeddinghaus@usb.ch (J.B.); max.wagener@usb.ch (M.W.); gregor.leibundgut@usb.ch (G.L.); christoph.kaiser@usb.ch (C.A.K.); 2 Department of General Internal Medicine, University Hospital Basel, 4031 Basel, Switzerland; 3 Department of Health Sciences and Technology, Swiss Federal Institute of Technology, 8093 Zurich, Switzerland

**Keywords:** aortic stenosis, TAVR, TAVI, LVEF

## Abstract

**Introduction**: Transcatheter aortic valve replacement (TAVR) has become an efficient and safe alternative to surgical aortic valve replacement (SAVR). While severe aortic stenosis as well as severe aortic regurgitation (AR) are known to negatively impact left ventricular ejection fraction (LVEF), prior studies have shown that TAVR can lead to an improvement in LVEF. Thus far, little is known about the prognostic implication of LVEF improvement as a sole predictor of outcomes. Therefore, the aim of this study was to assess the prognostic impact of LVEF impairment before TAVR, as well as early LVEF improvement in patients undergoing TAVR. **Materials and Methods**: Patients undergoing TAVR in a large tertiary university hospital were consecutively included in a prospective registry. Transthoracic echocardiography (TTE) was performed at baseline, after 1 month and annually thereafter. Significant LVEF improvement was defined as a relative increase of ≥10% in LVEF at 30 days compared to baseline LVEF. The primary outcome was all-cause mortality at 1 year. Secondary outcomes were major adverse cardiovascular events (MACEs) including cardiovascular death, non-fatal myocardial infarction, stroke, bleeding and unplanned re-interventions of the aortic valve at 5 years. **Results**: Among 1655 patients who underwent TAVR between September 2011 and April 2024, the LVEF at baseline was available for 1556 patients. Of these, 1031 patients (66.2%) had preserved LVEF at baseline (LVEF ≥ 53%), whereas 303 patients (19.5%) had moderately reduced LVEF (40–52%) and 222 patients (14.3%) had severely reduced LVEF (<40%). Out of the patients with impaired LVEF, 155 (40.4%) patients showed a significant improvement in LVEF ≥10% after 30 days, while 229 (60.6%) patients showed no significant LVEF improvement (<10%). Patients with preserved LVEF at baseline had significantly better mortality outcomes than those with severely reduced LVEF (*p* < 0.001). LVEF improvement was associated with a survival benefit after 1 year (*p* = 0.009, HR 2.68, 0.95 CI 1.23–5.85) which diminished after 5 years (*p* = 0.058), but patients with LVEF improvement showed lower MACE rates at 5 years (*p* < 0.001). **Conclusions**: Preserved LVEF before TAVR is an independent predictor for improved outcomes. Additionally, early improvement in LVEF is associated with beneficial outcomes in patients undergoing TAVR.

## 1. Introduction

Aortic stenosis (AS) is the most common valve pathology, affecting up to 20% of the population aged 75 and older [1]. Early detection and treatment are of high importance as untreated patients with severe aortic stenosis face high mortality and morbidity [2]. In recent years, transcatheter aortic valve replacement (TAVR) has become a viable alternative to surgical aortic valve replacement (SAVR), especially in elderly patients [3,4,5]. Recent guidelines by the European Society of Cardiology (ESC) support TAVR as the first-line treatment in patients older than 75 years irrespective of risk categories [6].

Aortic stenosis leads to numerous adaptations by the left ventricle. Narrowing of the aortic valve area leads to an increased afterload. This induces hypertrophy of the myocytes to maintain systemic cardiac output [7]. However, left ventricular hypertrophy itself imposes negative effects on ventricular function and coronary circulation, contributing to an increased rate of cardiovascular events [8]. Finally, these events lead to fibrosis of the myocytes, ventricular dilation and a decline in left ventricular ejection fraction (LVEF), and eventually to the development of heart failure [9].

Several studies have shown that valve replacement, whether by SAVR or TAVR, improves the LVEF in patients with pre-interventional left ventricular dysfunction [10]. Many factors have been shown to affect outcomes in patients undergoing TAVR, yet data on the impact of early LVEF improvement itself are scarce.

The objective of this study was to assess the rate of LVEF improvement after TAVR and to determine the prognostic impact of LVEF at baseline as well as early LVEF improvement in patients undergoing TAVR.

## 2. Methods

### 2.1. Study Design and Patient Cohort

All patients who underwent TAVR at the University Hospital Basel, Switzerland, were included in a prospective national database, as part of the Swiss TAVI registry, mandated by the Swiss health authorities (NCT01368250). The Swiss TAVI registry is a multicentric database and has been featured in recent publications [11,12,13]. The Swiss TAVI registry has been approved by the local cantonal ethics committee and the institutional review boards of all participating sites. All patients provided written informed consent for study participation and prospective follow-up assessment. The present study includes patients that underwent TAVR between September 2011 and April 2024.

### 2.2. Data Collection and Clinical Endpoints

All patient-related data, including baseline characteristics, procedural and follow-up information, were prospectively collected and recorded in a web-based database. Clinical follow-up data were obtained through standardized interviews, documentation from referring physicians and hospital discharge summaries. All adverse events were systematically collected and adjudicated by a dedicated clinical event committee.

We included all patients who had undergone TAVR and had transthoracic echocardiography (TTE) at baseline with measurements performed using the biplane Simpson method. In case of severely reduced examination conditions, LVEF was graded by visual analysis by treating physicians into the categories mentioned below. Patients were then divided into three separate groups based on their baseline LVEF. We defined patients with preserved LVEF (>52%), patients with moderately reduced LVEF (≥40%; ≤52%) and patients with severely reduced LVEF (<40%). We chose to defer from the definition of preserved ejection fraction defined by the European Society of Cardiology (ESC) due to the definitions in our local echocardiography department, where the transition between “preserved” and “borderline preserved” is defined at an LVEF of 53%. Follow-up TTE was either performed at our clinical echocardiographic laboratory or in cooperation with the patient’s treating cardiologists.

For analysis of LVEF improvement, patients were included if follow-up LVEF data after 30 days were available. Patients that had died before their first follow-up at day 30 were not considered for further analysis. Patients with moderately or severely reduced LVEF were categorized based on whether their LVEF had improved by more than 10% by day 30, later referred to as our cohort with “improved” patients, and patients with less than 10% LVEF improvement are referred to as “not-improved” patients. Patients with initially preserved LVEF were not considered for analysis of LVEF improvement.

Our primary endpoint was all-cause death at 1 year in patients with or without significant LVEF improvement. Secondary endpoints were all-cause death at 5 years and a combined endpoint of major adverse cardiovascular events (MACEs), defined as cardiovascular death, myocardial infarction, cerebrovascular stroke, bleeding and unplanned re-intervention of the aortic valve at 5 years. We also compared 1- and 5-year all-cause mortality between patients with preserved LVEF at baseline and patients with severely reduced LVEF as well as MACEs at 5 years.

### 2.3. Statistical Analysis

Categorical variables are presented as frequencies and percentages. Continuous variables are represented as mean values ± standard deviation if normal distribution was apparent. Non-normally distributed variables are represented as median values with the 25th and 75th quartiles. Comparisons between normally distributed continuous variables were performed using Fisher’s paired *t*-test for normally distributed variables. Non-normally distributed variables were compared using the Mann–Whitney U test. Differences between ordinal scaled data were calculated using Pearson’s Chi^2^ test. Survival analysis was carried out using the Kaplan–Meier procedure, and differences in survival were tested with the Log-Rank (Mantel–Cox) test. A *p*-value < 0.05 was considered significant. We used multivariate logistic regression models to determine factors with an impact on LVEF improvement. All statistical analyses were performed using R 4.2.3 (R Foundation for Statistical Computing, Vienna, Austria). Sankey plots were created using the open-source tool SANKEYMATIC by Steve Bogart, available at https://www.sankeymatic.com (accessed on 20 January 2024).

## 3. Results

### 3.1. Patient Cohort and Baseline Characteristics

Among 1655 patients that underwent TAVR between September 2011 and April 2024, the TTE at baseline with valuable LVEF measurements was available in 1556 patients (94.0%). At baseline, 525 of 1556 patients (33.7%) were diagnosed with impaired LVEF. Out of these, follow-up TTE data including LVEF measurements were available in 384 (73.1%) cases.

Among patients with impaired LVEF at baseline, 303 (57.7%) patients had moderately reduced LVEF and 222 (42.3%) had severely reduced LVEF. Table 1 summarizes the baseline data for patients with preserved (>52%), moderately reduced (40–52%) and severely reduced (≤40%) LVEF. Table 2 shows interventional and echocardiographic findings. Compared to patients with preserved LVEF at baseline, patients with severely reduced LVEF were more often male (66.7% vs. 46.9%, *p* < 0.001); had more comorbidities such as coronary artery disease (CAD) (64.9% vs. 50.0%, *p* < 0.001), prior myocardial infarction (31.5% vs. 11.9%, *p* < 0.001) and diabetes (37.8% vs. 26.3%, *p* = 0.002) and had more often undergone interventions such as prior percutaneous coronary intervention (PCI) (38.3% vs. 30.2%, *p* < 0.001), cardiac surgery of any kind (15.8% vs. 7.5%, *p* < 0.001) or pacemaker implantation prior to TAVR (17.1% vs. 7.5%, *p* < 0.001). Patients with severely reduced LVEF were more frequently diagnosed with left ventricular hypertrophy (20.7% vs. 7.6%, *p* < 0.001). Additionally, they showed lower peak aortic valve gradients (49.0 vs. 70.0, *p* < 0.001), lower mean gradients (33 ± 13.8 vs. 45 ± 13.5, *p* = 0.001) and more atrial fibrillation (AF) (30.6% vs. 23.4%, *p* = 0.03). In terms of access site, no differences were found. In patients with moderately or severely reduced LVEF, valve sizes for TAVR were slightly larger than in patients with preserved LVEF (27.0 vs. 26.0, *p* < 0.001).

#### LVEF Improvement

Among 384 patients with moderately or severely reduced LVEF at baseline, 155 patients (40.4%) showed LVEF improvement after 30 days, while 229 (60.6%) patients showed no improvement. Mean LVEF improvement and decline in all patients was 7.8% (±9.67). Among the latter, 68 patients (26.6%) showed a decline in LVEF. Table 3 summarizes these groups’ baseline data, and Table 4 gives insights on echocardiographic and interventional findings. Patients with LVEF improvement compared to those without were more often female (47.7% vs. 32.3%, *p* = 0.003) and had less frequently undergone prior cardiac surgery of any kind (9.0% vs. 16.6%, *p* = 0.049). There was a difference in baseline LVEF between these groups, with baseline LVEF being significantly lower in patients that would show improvement after 30 days (38.0% vs. 42.0%, *p* = 0.002). Additionally, patients with LVEF improvement showed a smaller indexed aortic valve area (0.2 cm^2^/m^2^ vs. 0.3 cm^2^/m^2^, *p* = 0.03) and higher peak aortic valve gradients (60 mmHg vs. 53 mmHg, *p* = 0.011) and mean gradients (41.0 mmHg vs. 38.0 mmHg, *p* = 0.049). Right ventricular dysfunction was significantly less frequent in patients with LVEF improvement (14.2% vs. 31%, *p* < 0.001). Relevant medications are listed in the Supplementary Table S1.

Left ventricular hypertrophy at baseline was numerically, albeit insignificantly, less frequent in patients that showed improvement than in patients without improvement (16.7% vs. 20.9%, *p* = 0.37). After 30 days, left ventricular hypertrophy prevalence was declining in both groups but was significantly less frequent in patients with LVEF improvement (3.2% vs. 12.2%, *p* = 0.003). There was a difference regarding access site for TAVR, with transapical access being less frequent in patients with LVEF improvement (5.1% vs. 13.2%, *p* = 0.003). Multivariate linear regression for several factors such as age, sex, CAD, CKD, prior PCI, prior heart surgery, diabetes, hypertension, pacemaker implantation and cerebrovascular disease showed no predictive value for LVEF improvement at 30 days (Table 5).

**Figure 1:**
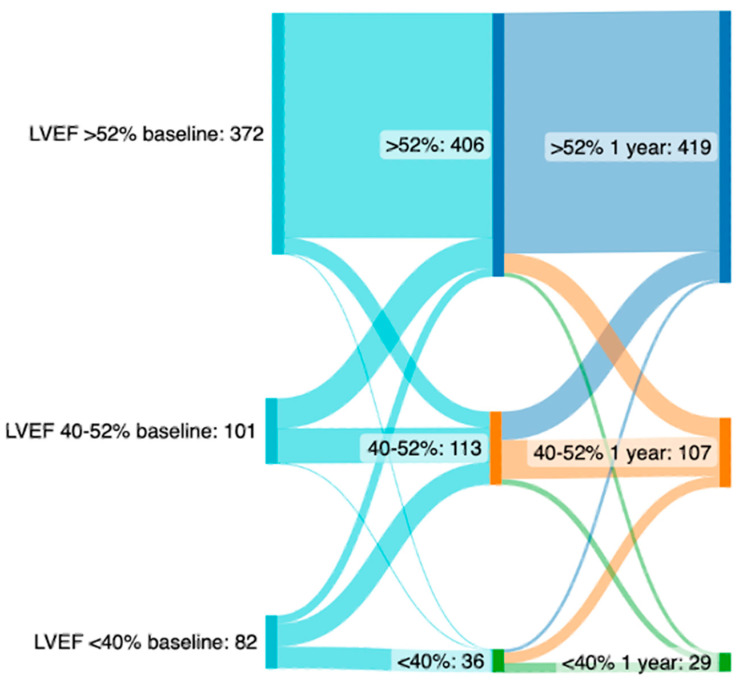
Sankey plot depicting the course of LVEF in patients with severely reduced, moderately reduced and preserved LVEF at baseline (**left**), 30 days (**middle**) and after 1 year (**right**).

The overall dynamics of LVEF change after TAVR are depicted in Figure 1. After 1 year, LVEF improvement had been maintained in the vast majority of patients (preserved LVEF at 30 days, 72%; after 1 year, 76%).

### 3.2. Outcomes after TAVR

**Figure 2:**
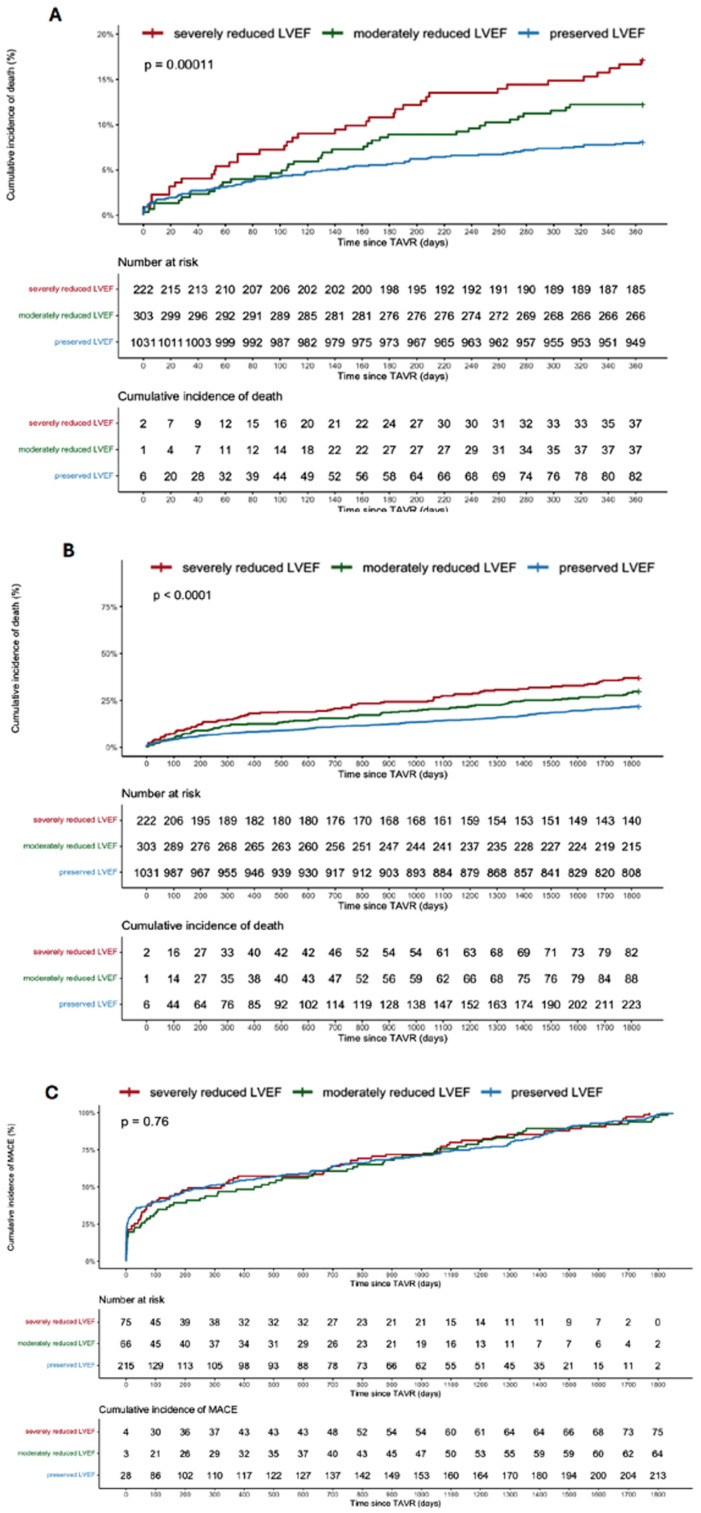
(**A**–**C**) Kaplan–Meier curves showing mortality after 1 year (**A**) and at 5 years (**B**) as well as MACEs at 5 years (**C**) in patients with preserved (LVEF > 52%), moderately reduced (40–52%) and severely reduced (LVEF < 40%) baseline LVEF.

Follow-up data were available in 88.9% of patients at 1 year and in 35.7% of patients at 5 years. Mortality after one year was lower in patients with preserved LVEF at baseline compared to patients with severely reduced LVEF (7.9% vs. 16.6%, *p* < 0.001, HR 0.45, 0.95 CI 0.30–0.66), as was mortality after five years (21.6% vs. 36.9%, *p* < 0.001, HR 0.52, 0.95 CI 0.40–0.67). There was no difference in the frequency of MACEs after five years (213 vs. 75 events, *p* = 0.76). The outcomes are depicted in Figure 2A–C.

**Figure 3:**
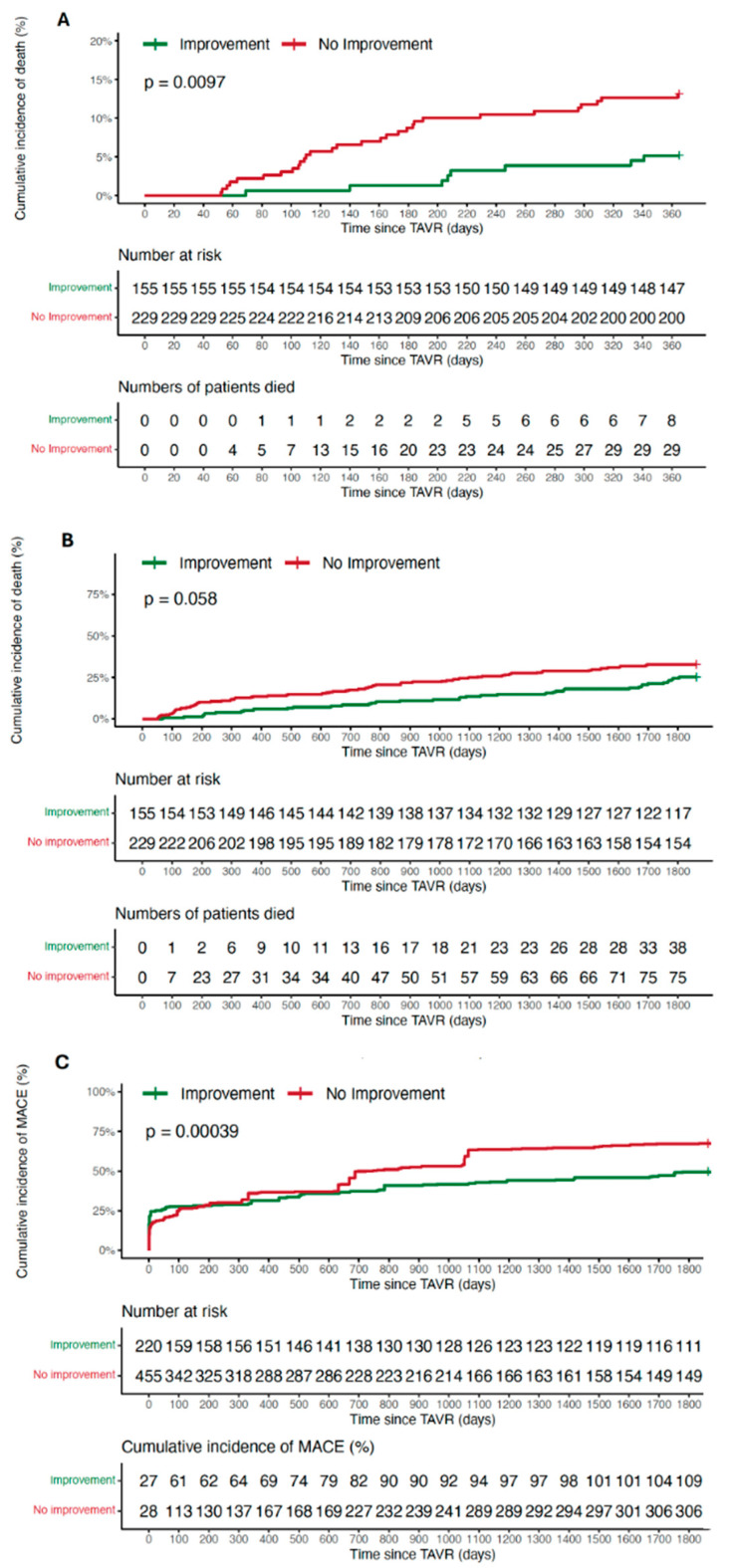
(**A**–**C**) Kaplan–Meier curves showing mortality in patients with ≥10% and <10% LVEF improvement after one (**A**) and five (**B**) years, as well as MACEs at five years (**C**).

Patients with LVEF improvement showed significantly lower mortality after one year (5.2% vs. 12.6%, *p* = 0.009, HR 2.68, 0.95 CI 1.23–5.85). There was a numerical, albeit insignificant, difference after five years (24.5% vs. 32.7%, *p* = 0.058, HR 1.45, 0.95 CI 0.98–2.13). Additionally, the combined endpoint of MACEs after five years occurred less frequently (109 vs. 306 events, *p* < 0.001). The outcomes are depicted in Figure 3A–C.

## 4. Discussion

In this study, we aimed to analyze the role of LVEF impairment and early improvement in LVEF in patients undergoing TAVR in a tertiary university center.

In the present study, patients showing improvements in LVEF were more often female than patients without improvement. Studies have shown that males generally suffer more frequently from ischemic heart disease than females, which might lead to a lower potential for LVEF recovery. In their systematic review, Kewcharoen et al. found that female gender is associated with a higher chance of LVEF recovery [14]. Other studies have also displayed gender-specific differences in left ventricular geometry as well as the extent of myocardial fibrosis [14,15], which may explain the differences in the reversal of left ventricular hypertrophy and left ventricular function after TAVR. Supporting these findings, Chen et al. showed that female patients have favorable reversed left ventricular remodeling, with earlier and greater LV mass regression [16]. These factors may contribute to higher chances of LVEF improvement in female patients.

**Figure 4:**
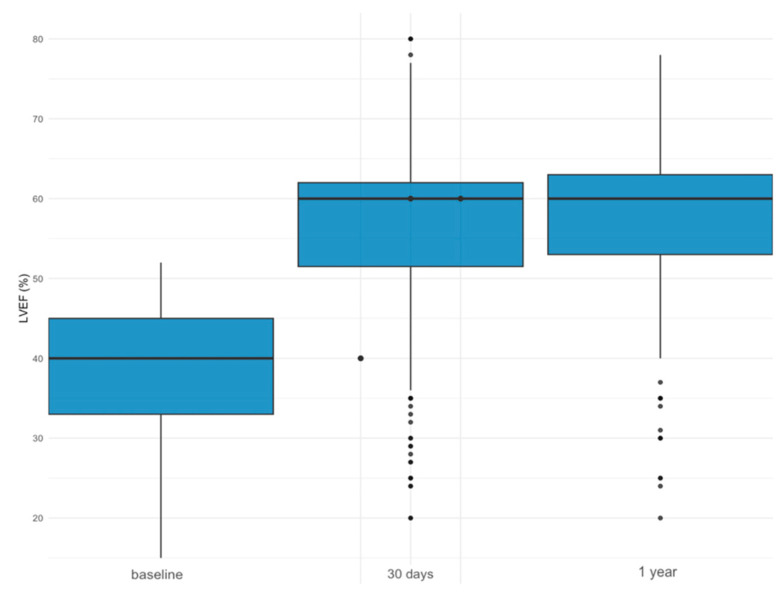
Evolution of LVEF in patients with LVEF ≤ 52% at baseline, after 30 days and after 1 year.

Our study showed that in most patients with moderately or severely reduced LVEF, TAVR can lead to an improvement in LVEF, as depicted in Figure 4. This effect was constant in both patients with moderately reduced and those with severely reduced LVEF and was maintained after 1 year. Our findings underline the positive impact of TAVR on left ventricular dimensions, especially with left ventricular hypertrophy declining over the course of one year. It is known that high-grade aortic stenosis leads to chronic pressure overload due to high transaortic valve gradients, leading to pathologic remodeling in the form of concentric hypertrophy [17]. Correcting the high-pressure gradient using TAVR leads to reduced pressure overload for the left ventricle and might partly explain the improvement in left ventricular function that some, yet not all, patients achieve after TAVR [18]. Changes in left ventricular dimensions differed after TAVR, with the LVEDD being smaller and left ventricular hypertrophy being less frequent in patients with LVEF improvement. These changes support the recline of left ventricular remodeling after TAVR, contributing to the improvement of left ventricular function. TAVR aims to treat one of the causes of impaired EF. However, heart failure in patients with severe AS is not always caused by the stenosis alone. The vast majority of patients have concomitant cardiovascular diseases; therefore, medical heart failure treatment plays a major role in these patients as well. Especially younger patients with reduced ejection will benefit from long-term guideline-directed medical therapy and regular follow-up after TAVR to maintain or restore an impaired EF [19].

We found a difference in mortality after one year between patients with and without LVEF improvement, but only a numeric, statistically insignificant difference after 5 years. Contrary to our findings, Kolte et al. showed that an LVEF improvement of more than 10% is associated with decreased overall and cardiac death at 5 years [20]. We were not able to identify predictive factors associated with poor LVEF improvement, which may be explained by our sample size not being powered for this matter. However, the findings of Kuneman et al. demonstrated an association of CAD, prior myocardial infarction and permanent pacemaker implantation prior to TAVR with worse or missing LVEF improvement [21].

Additionally, our study revealed significant differences in mortality between patients with preserved LVEF at baseline compared to patients with impaired LVEF after one and five years. An explanation for this finding could be the fact that comorbidities such as CAD and prior cardiac surgery were more frequent in patients with impaired LVEF. Nevertheless, aortic valve stenosis itself negatively influences LV function and outcomes. Patients with an initial decline in their LVEF with severe aortic valve stenosis might benefit from earlier aortic valve replacement to prevent further deterioration of LV function [22]. Moreover, Dauerman and colleagues demonstrated that favorable 1-year outcomes were linked to early improvement in left ventricular ejection fraction (LVEF) [23].

In our study, the incidence of MACEs was less frequent in patients with significant LVEF improvement compared to patients without LVEF improvement. These findings are in contrast with previous research by Kuneman et al., who showed no difference in long-term event-free survival in patients with >5% improvement in LVEF compared to <5% improvement [21].

Our findings contribute to the current body of evidence on long-term outcomes after aortic valve replacement, particularly in the context of an increasing number of patients undergoing TAVR. We report the five main findings below.

First, patients with severely or moderately reduced LVEF at baseline were more often male and had a higher prevalence of comorbidities. They also presented more frequently with left ventricular hypertrophy, lower mean gradients and atrial fibrillation. Second, while 155 (40.4%) patients with severely or moderately reduced LVEF at baseline showed an improved LVEF after 30 days, 229 (60.6%) showed no improvement, and of these, 58 (25.3%) even showed a decline. Third, despite impaired LVEF function at baseline being more common in male patients, female patients more often had an LVEF improvement after 30 days (47.7% vs. 32.3%, *p* = 0.003). Fourth, patients with LVEF improvement at 30 days maintained their improvement after 1 year, highlighting a prolonged beneficial effect of TAVR on LVEF improvement. Lastly, we noted that improvement in LVEF was associated with improved mortality after one year and was linked to a reduction in MACEs after five years. Specifically, among the MACE endpoints, the occurrence of major bleeding and cardiovascular death appeared to be the most significantly associated with LVEF improvement.

Our study has several notable limitations. Firstly, the absence of uniformity in the laboratories conducting follow-up transthoracic echocardiographic studies is a limitation; however, our study represents real world data. Since our patients typically attended follow-up visits with external cardiologists, variations in the investigation protocols and results may arise based on the investigator. Additionally, given the retrospective nature of this data analysis, our study lacks the statistical power required to demonstrate clear differences in mortality outcomes. To ascertain the benefits of event-free survival conclusively, larger studies with a higher sample size may be necessary. Our study reflects real world data from a large tertiary university hospital over more than 10 years with consecutively enrolled patients in a prospective registry.

## 5. Conclusions

In conclusion, TAVR led to sustained LVEF improvement after 30 days and 1 year. Patients with preserved LVEF at baseline experienced better long-term outcomes. In addition, those who showed early improvement in LVEF post-TAVR had lower mortality rates after 1 year and a reduction in mortality and fewer cardiovascular events at 5 years. Further research is needed to elucidate the precise mechanisms impeding LVEF improvement.

## Figures and Tables

**Table 1:** Baseline characteristics of patients with preserved (>52%), moderately reduced (40–52%) and severely reduced (<40%) LVEF.

	Severely Reduced LVEF (*n* = 222)	Moderately Reduced LVEF (*n* = 303)	Preserved LVEF (*n* = 1031)	*p*
Sex				
Female	74 (33.3%)	129 (42.6%)	547 (53.1%)	<0.001
Male	148 (66.7%)	174 (57.4%)	484 (46.9%)	
Age (years)	82.8 [77.9, 87.0]	83.5 [79.6, 87.4]	82.5 [78.8, 85.9]	0.065
BMI (kg/m^2^)	25.1 [23.0, 28.5]	25.6 [22.9, 28.7]	26.3 [23.4, 29.6]	0.013
EuroScore II	4.9 [2.8, 8.6]	3.2 [1.8, 5.4]	1.9 [1.2, 3.5]	<0.001
Society of Thoracic Surgeons (STS) Score	4.2 [2.6, 6.7]	3.9 [2.5, 6.7]	3.2 [2.2, 5.3]	<0.001
Hypertension	173 (77.9%)	247 (81.5%)	832 (80.7%)	0.560
Diabetes	84 (37.8%)	80 (26.4%)	271 (26.3%)	0.002
Dyslipidemia	117 (52.7%)	181 (59.7%)	628 (60.9%)	0.077
Coronary artery disease (CAD)	144 (64.9%)	200 (66.0%)	515 (50.0%)	<0.001
Prior myocardial infarction (MI)	70 (31.5%)	66 (21.8%)	123 (11.9%)	<0.001
Prior percutaneous coronary intervention (PCI)	85 (38.3%)	136 (44.9%)	311 (30.2%)	<0.001
Prior aortic valvuloplasty	15 (6.8%)	10 (3.3%)	28 (2.7%)	0.011
Prior cardiac surgery	35 (15.8%)	46 (15.2%)	77 (7.5%)	<0.001
Prior implanted cardiac pacemaker	38 (17.1%)	39 (12.9%)	83 (8.1%)	<0.001
Implanted cardiac defibrillator (ICD)	4 (1.8%)	1 (0.3%)	4 (0.4%)	0.034
Atrial fibrillation (AF)	68 (30.6%)	85 (28.1%)	241 (23.4%)	0.037
Cerebrovascular disease (Stroke/TIA)	27 (12.2%)	38 (12.5%)	128 (12.4%)	0.991
Peripheral artery disease (PAD)	46 (20.7%)	60 (19.8%)	160 (15.5%)	0.066
Chronic kidney disease (CKD)	170 (77.6%)	217 (72.1%)	609 (59.2%)	<0.001
Dialysis	10 (4.5%)	9 (3.0%)	18 (1.7%)	0.038
COPD	16 (7.2%)	32 (10.6%)	95 (9.2%)	0.421

Abbreviations. BMI: body mass index; BSA: body surface area; STS: Society of Thoracic Surgeons; CKD: chronic kidney disease; GFR: glomerular filtration rate; CAD: coronary artery disease; PCI: prior percutaneous intervention; COPD: chronic obstructive pulmonary disease; ICD: implanted cardioverter defibrillator.

**Table 2:** Baseline echocardiographic and interventional findings of patients with preserved (>52%), moderately reduced (40–52%) and severely reduced (<40%) LVEF.

	Severely Reduced LVEF (*n* = 222)	Moderately Reduced LVEF (*n* = 303)	Preserved LVEF (*n* = 1031)	*p*
LVEF at baseline (%)	31.0 [27.0, 35.0]	45.0 [42.0, 50.0]	60.0 [58.0, 65.0]	<0.001
Left ventricular hypertrophy at baseline	46 (20.7%)	54 (17.8%)	79 (7.6%)	<0.001
Aortic valve area (cm^2^)	0.7 [0.6, 0.8]	0.7 [0.6, 0.9]	0.8 [0.6, 0.9]	<0.001
Indexed aortic valve area (cm^2^/m^2^)	0.3 [0.2, 0.3]	0.3 [0.2, 0.3]	0.3 [0.2, 0.3]	<0.001
Peak aortic valve gradient (mmHg)	49.0 [36.0, 68.0]	61.0 [44.0, 76.0]	70.0 [54.0, 83.0]	<0.001
Mean aortic valve gradient (mmHg)	34.0 [25.0, 43.0]	42.0 [34.0, 52.0]	45.0 [40.0, 53.5]	<0.001
Right ventricular dysfunction at baseline	72 (35.6%)	64 (23.5%)	51 (5.6%)	<0.001
Pulmonary hypertension at baseline	46 (22.8%)	54 (19.9%)	79 (8.6%)	<0.001
Left ventricular mass (g)	228.0 [191.0, 256.5]	204.0 [175.0, 238.0]	185.0 [150.0, 223.0]	<0.001
Indexed left ventricular mass (g/m^2^)	123.5 [107.0, 141.0]	116.5 [96.0, 139.0]	104.0 [88.0, 122.0]	<0.001
Left ventricular end-diastolic diameter (LVEDD, mm)	54.0 [48.0, 58.0]	48.0 [43.0, 53.0]	44.0 [39.0, 48.0]	<0.001
Left ventricular end-systolic diameter (LVESD, mm)	45.0 [38.0, 49.0]	35.0 [30.0, 40.0]	28.0 [24.0, 33.0]	<0.001
Valve prothesis size (mm)	27.0 [26.0, 29.0]	27.0 [25.0, 29.0]	26.0 [25.0, 29.0]	<0.001
Expandable valve type				
Mechanical-expandable	6 (2.7%)	21 (6.9%)	57 (5.6%)	0.001
Self-expandable	129 (58.4%)	186 (61.4%)	702 (68.4%)	
Balloon-expandable	86 (38.9%)	96 (31.7%)	268 (26.1%)	
Vascular access for TAVR				0.377
Transfemoral access	192 (86.5%)	253 (83.5%)	911 (88.4%)	
Transapical access	23 (10.4%)	40 (13.2%)	94 (9.2%)	
Subclavian access	6 (2.7%)	8 (2.6%)	24 (2.3%)	
Direct aortic access	1 (0.4%)	2 (0.7%)	2 (0.1%)	

Abbreviations. AVA: aortic valve area; LVEF: left ventricular ejection fraction; LVEDD: left ventricular end-diastolic diameter; LVESD: left ventricular end-systolic diameter; Massindex: left ventricular mass index; AV max: maximum aortic valve pressure gradient; AV mean: mean aortic valve pressure gradient; hypertrophy: left ventricular hypertrophy.

**Table 3:** Baseline characteristics of patients showing significant LVEF improvement (≥10%) at 30-day follow-up (“Improvement”) vs. patients without significant (<10%) LVEF improvement (“No Improvement”).

	Improvement (*n* = 155)	No Improvement (*n* = 229)	*p*
Sex			
Female	74 (47.7%)	74 (32.3%)	0.003
Male	81 (52.3%)	155 (67.7%)	
Age	82.5 (±6.6)	82.1 (±6.9)	0.496
BMI	25.5 [23.0, 28.4]	26.4 [23.4, 29.1]	0.316
EuroScore II	4.1 [2.0, 5.8]	3.7 [2.0, 6.0]	0.857
Society of Thoracic Surgeons (STS) Score	4.0 [2.6, 6.5]	3.7 [2.4, 6.3]	0.583
Hypertension	119 (76.8%)	188 (82.1%)	0.251
Diabetes	49 (31.6%)	69 (30.1%)	0.845
Dyslipidemia	78 (50.3%)	132 (57.6%)	0.190
Coronary artery disease (CAD)	95 (61.3%)	155 (67.7%)	0.238
Prior myocardial infarction (MI)	40 (25.8%)	52 (22.7%)	0.564
Prior percutaneous coronary intervention (PCI)	61 (39.4%)	101 (44.1%)	0.413
Prior aortic valvuloplasty	3 (1.9%)	15 (6.6%)	0.064
Prior cardiac surgery	14 (9.0%)	38 (16.6%)	0.049
Prior implanted cardiac pacemaker	19 (12.3%)	41 (17.9%)	0.176
Implanted cardiac defibrillator (ICD)	1 (0.6%)	2 (0.9%)	1.000
Atrial fibrillation (AF)	37 (23.9%)	74 (32.3%)	0.094
Cerebrovascular disease (Stroke/TIA)	19 (12.3%)	23 (10.0%)	0.606
Peripheral artery disease (PAD)	24 (15.5%)	46 (20.1%)	0.312
Chronic kidney disease (CKD)	105 (68.2%)	168 (74.3%)	0.233
Dialysis	3 (1.9%)	11 (4.8%)	0.233
COPD	9 (5.8%)	20 (8.7%)	0.385

Abbreviations. BMI: body mass index; BSA: body surface area; STS: Society of Thoracic Surgeons; CKD: chronic kidney disease; GFR: glomerular filtration rate; CAD: coronary artery disease; PCI: prior percutaneous intervention; COPD: chronic obstructive pulmonary disease; ICD: implanted cardioverter defibrillator.

**Table 4:** Baseline echocardiographic findings and interventional characteristics of patients with significant (≥10%) LVEF improvement at 30 days follow-up (“Improvement”) vs. patients without significant (<10%) LVEF improvement (“No Improvement”).

	Improvement (*n* = 155)	No Improvement (*n* = 229)	*p*
LVEF at baseline (%)	38.0 [30.0, 44.5]	42.0 [35.0, 47.0]	<0.001
Left ventricular hypertrophy at baseline	26 (16.7%)	48 (20.96%)	0.37
Aortic valve area (cm^2^)	0.7 [0.5, 0.8]	0.8 [0.6, 0.9]	0.008
Indexed aortic valve area (cm^2^/m^2^)	0.2 [0.2, 0.3]	0.3 [0.2, 0.3]	0.033
Peak aortic valve gradient (mmHg)	60.0 [45.0, 78.0]	53.0 [37.0, 71.0]	0.011
Mean aortic valve gradient (mmHg)	41.0 [31.0, 50.1]	38.0 [27.0, 48.0]	0.049
Right ventricular dysfunction at baseline	22 (14.2%)	71 (31.0%)	<0.001
Pulmonary hypertension at baseline	26 (18.6%)	48 (23.9%)	0.300
Left ventricular mass (g)	225.5 [189.0, 256.0]	218.0 [178.5, 250.5]	0.428
Indexed left ventricular mass (g/m^2^)	124.5 [112.2, 140.0]	114.0 [98.0, 140.0]	0.101
Left ventricular end-diastolic diameter (LVEDD, mm)	50.0 [44.0, 54.2]	50.0 [45.0, 56.0]	0.242
Left ventricular end-systolic diameter (LVESD, mm)	38.0 [33.0, 45.0]	39.0 [32.0, 45.0]	0.965
Valve prothesis size (mm)	26.0 [25.0, 29.0]	27.0 [26.0, 29.0]	0.016
Expandable valve type			0.405
Mechanical-expandable	8 (5.2%)	13 (5.6%)	
Self-expandable	88 (56.8%)	144 (62.9%)	
Balloon-expandable	59 (38.0%)	72 (31.4%)	
Vascular access for TAVR			0.042
Transfemoral access	143 (92.3%)	198 (86.5%)	
Transapical access	7 (4.5%)	27 (11.8%)	
Subclavian access	5 (3.2%)	3 (1.3%)	
Direct aortic access	0	1 (0.4%)	

Abbreviations. AVA: aortic valve area; LVEF: left ventricular ejection fraction; LVEDD: left ventricular end-diastolic diameter; LVESD: left ventricular end-systolic diameter; Massindex: left ventricular mass index; AV max: maximum aortic valve pressure gradient; AV mean: mean aortic valve pressure gradient; hypertrophy: left ventricular hypertrophy.

**Table 5:** Multivariable logistic regression analysis for common comorbidities as predictors of LVEF improvement.

Variable	R^2^	*p*
Age	0.06	0.64
Sex	<0.001	0.34
Arterial hypertension	0.008	0.07
Diabetes	0.002	0.31
CKD	0.003	0.29
CAD	0.001	0.49
Prior PCI	0.002	0.42
Prior heart surgery	0.003	0.28
Prior myocardial infarction	<0.005	0.75
Atrial fibrillation	0.002	0.49
Cerebrovascular disease	0.006	0.10
Implanted pacemaker	0.013	0.02

## Data Availability

The data presented in this study are available on request from the corresponding author.

## Supplementary Materials

The following supporting information can be downloaded at: https://www.mdpi.com/article/10.3390/jcm13133639/s1, Table S1: Baseline medication before TAVR in patients with or without early LVEF-improvement.

## Abbreviations

AS	Aortic stenosis
CAD	Coronary artery disease
CABG	Coronary arterial bypass graft
CCS-Score	Canadian Cardiovascular Society-Score
CKD	Chronic kidney disease
CVD	Cerebrovascular disease
eGFR	Estimated glomerular filtration rate
ESC	European Society of Cardiology
EuroSCORE	European System for Cardiac Operative Risk Evaluation
ICD	Implanted cardioverter defibrillator
LVEF	Left ventricular ejection fraction
LVEDD	Left ventricular end-diastolic diameter
LVESD	Left ventricular end-systolic diameter
NYHA	New York Heart Association
PCI	Percutaneous coronary intervention
STS	Society of Thoracic Surgeons
SAVR	Surgical aortic valve replacement
TAVR	Transcatheter aortic valve replacement
TTE	Transthoracic echocardiography
R^2^	Coefficient of determination indicating correlation, where a value ≤ 0.35 indicates low or weak correlation
