# Supplementary material for: Prognostic Impact of Left Ventricular Ejection Fraction Improvement after Transcatheter Aortic Valve Replacement
Source: J Clin Med. 2024 Jun 21;13(13):3639. doi: 10.3390/jcm13133639 (PMC11242474; doi:10.3390/jcm13133639)
Supplement: Supplementary file 1 [file jcm-13-03639-s001.zip › jcm-3053806-supplementary.pdf]

Article

Prognostic impact of left ventricular ejection fraction improvement after transcatheter aortic valve replacement

Jakob Reichl [1,2*]; Thorald Stolte [1,3*]; Shihui Tang [1]; Jasper Boeddinghaus [1]; Max Wagener [1], Gregor Leibundgut [1], Christoph Kaiser [1] and Thomas Nestelberger [1,*]

Table S1. Baseline medication before TAVR in patients with or without early LVEF-improvement.

	Improvement (n = 155)	No Improvement (n = 229)
**ACE-Inhibitor**	52 (33.5%)	76 (33.2%)
**AT1-Inhibitor**	41 (26.5%)	54 (23.6%)
**Sacubitril/Valsartan**	3 (1.9%)	9 (3.9%)
**Betablocker**	96 (61.9%)	145 (63.3%)
**SGLT-2-Inhibitor**	4 (2.5%)	5 (2.2%)
**Calcium-antagonist***	24 (15.5%)	29 (12.6%)
**Diuretic therapy**	117 (75.5%)	208 (75.5%)

* Dihydropyridine-type
